# Food allergic teens: education, anaphylaxis and concerns

**DOI:** 10.1186/1710-1492-10-S2-A44

**Published:** 2014-12-18

**Authors:** NL Ross, CA Gillespie, CR Unruh, AB Becker

**Affiliations:** 1 Children’s Allergy & Asthma Education Centre, Health Sciences Centre, Winnipeg, Manitoba, R3E 0Z2, Canada; 2 University of Manitoba, Winnipeg, Manitoba, R3E 0W2, Canada

## Background

Adolescents with food allergy are at particular risk for life threatening anaphylaxis. Management of food allergies includes preventing, recognizing and responding to reactions. Focus groups were held as a preliminary step in the planning and development of effective education for this group.

## Methods

Focus groups were held from January to April 2014. Semi structured interviews were conducted to gather information about what teens with food allergy needed to know and how they like to learn. Interviews were digitally recorded, transcribed and reviewed.

## Results

16 adolescents (M = 11, F = 5); age 12-19; 15/16 peanut allergic, 10/16 other food allergens

All had epinephrine auto-injectors (EpiPen = 11; Allerject = 5)

Teens believe they are well informed; often from parents; however they did identify important topics to incorporate into an education program for teens. Teens need/want to learn more about: cross-contamination, advisory statements on food labels, allergens in non-food products, recognizing a reaction, staying calm during a reaction, teaching friends – signs of a reaction and auto-injector use, communicating confidently with others – strategies for what to say in situations and hands on practice with the auto-injectors.

Food allergy related topics teens would like to discuss: travelling, dating, partying, grocery shopping, cooking, symptoms of a reaction versus anxiety, new treatments and research.

Concerning themes around anaphylaxis noted were: reactions are dealt with by “waiting it out” or “sleeping it off”; epinephrine is only used if you can’t breathe, can’t talk or think you’re dying); antihistamines can be used as first line treatment.

## Conclusion

This study highlights knowledge gaps that exist around anaphylaxis in this group and identifies important topics to incorporate into a program. An education program delivered in an effective manner for adolescents that addresses these gaps and provides strategies to help manage food allergy may be helpful and increase confidence for this high-risk group.

